# Effects of Hot Extrusion Temperature Conditions on the Hardness and Electrical Conductivity of Rapidly Solidified Al-Fe Alloys

**DOI:** 10.3390/ma16145050

**Published:** 2023-07-17

**Authors:** Ryohei Kobayashi, Tatsuya Funazuka, Toru Maeda, Tomomi Shiratori

**Affiliations:** 1 Advanced Materials Laboratory, Sumitomo Electric Industries, Ltd., Itami-shi 664-0016, Japan; 2 Graduate School of Science and Engineering, University of Toyama, Toyama-shi 930-8555, Japan; 3 Academic Assembly Faculty of Engineering, University of Toyama, Toyama-shi 930-8555, Japan

**Keywords:** aluminum alloy, hot extrusion, electrical conductor, powder metallurgy, mechanical property

## Abstract

Rapidly solidified Al-Fe alloys produced by hot extrusion are a promising replacement for copper-based electrical conductors because of their light weight. However, the effects of the extrusion temperature conditions on the mechanical and electrical properties of extruded materials are unknown. The present work investigated the effects of billet preheating temperature, in situ temperature during extrusion, and additional heat treatment after extrusion on hardness and electrical conductivity. An air-jet atomized Al-2.3%Fe alloy powder was pre-sintered into cylindrical billets and then hot-extruded. The hardness of the extrudates decreased as the in situ temperature during extrusion increased above 650 K. The billet preheating temperature affected the in situ temperature during extrusion. Additional annealing after extrusion decreased the hardness. The cause of the decrease in hardness was coarsening of the grain of the aluminum matrix. The electrical conductivity increased with higher billet preheating temperatures before extrusion or additional annealing after extrusion; however, an in situ temperature rise for a few seconds during extrusion did not affect the conductivity. The increase in electrical conductivity was considered to be caused by a decrease in the amount of solute iron, which requires holding the material at a high temperature for longer than several minutes.

## 1. Introduction

**Figure 1:**
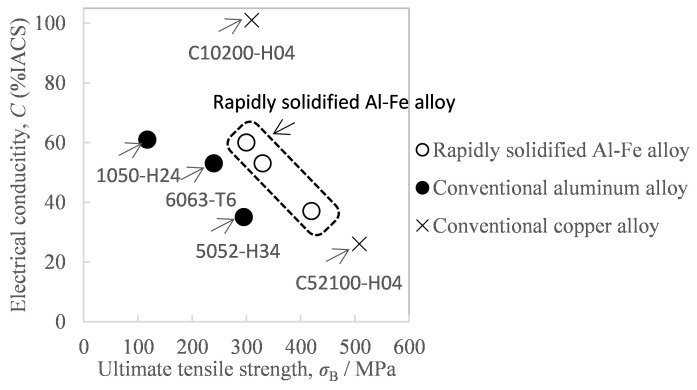
Comparison of the tensile strength and electrical conductivity of aluminum alloys and copper alloys. The property of the rapidly solidified Al-Fe alloy was cited from Reference [4].

With the recent growth in the types and number of smartphones and other mobile communication devices, as well as edge devices, it is now becoming increasingly important to reduce device weight in order to improve portability. From this viewpoint of weight reduction, aluminum, which has two-times greater strength and electrical conductivity per unit mass than conventional copper and requires half the mass to have equivalent strength and electrical conductivity, shows promise as a conductive material to replace copper [1]. However, aluminum materials with higher strength are in demand because contact resistance keeps increasing due to creep and plastic deformation when the materials are used in high temperatures such as connectors for high-current circuits [2]. Against this background, a rapidly solidified Al-Fe alloy has recently attracted attention as a conductive material to replace copper [3,4]. As shown in Figure 1, the rapidly solidified Al-Fe alloy has the combined benefits of high strength, heat resistance, and high conductivity [4]. Since such conductive materials are often used in the form of strips or wire rods, it is more favorable to make the product length greater so that the transmission distance can be increased. This rapidly solidified Al-Fe alloy can be obtained via powder metallurgy, by which the molten Al-Fe alloy is quenched into a powder, and the resultant powder is solidified by being compressed and then sintered. Its sintering methods include spark plasma sintering [5], hot pressing [6], additive manufacturing [7], and hot extrusion [3], but the most suitable method for obtaining long sintered bodies is hot extrusion. The hot extrusion method for aluminum alloy powder is a technique by which powder is pre-solidified by cold forming or such to make a billet, which is preheated and fed into a high-temperature mold called a container to be pressurized, and is then passed through a die with holes of certain desired shapes to form bars or certain shapes.

The rapidly solidified Al-Fe alloy has high strength and heat resistance because the Al-Fe intermetallic compounds are finely dispersed in the aluminum matrix, and this contributes to particle dispersion strengthening [4]. As for conductivity, this alloy is highly conductive because the solubility limit of iron to aluminum is low, less than 0.05% [8] (mass fraction; the same applies hereafter), and this helps maintain the aluminum matrix at high purity. The Al-Fe intermetallic compounds contained in Al-Fe alloys undergo a phase change from a metastable Al_6_Fe phase to a stable Al_13_Fe_4_ (sometimes referred to as Al_3_Fe) phase via heating at 623–673 K or above [7]. Since 623–673 K is close to the hot extrusion temperature [3], the extrusion temperature conditions can affect the mechanical and electrical properties of the extrudate. In general, the in situ temperature of billets rises due to the processing heat and container–billet friction [9,10]. Therefore, this rise in temperature can affect the properties; however, there are so far no studies on the effect. In addition, the effect of additional heat treatment at 673 K or above after extrusion on the electrical conductivity of the extrudates is unknown.

As described so far, the effects of the extrusion temperature conditions on the mechanical and electrical properties of a rapidly solidified Al-Fe alloy are of industrial importance since they affect the yield of the extrudate. On the other hand, the effect of these extrusion conditions and their mechanisms are not sufficiently understood. This is why we conducted the present study to understand the relationship between the extrusion temperature conditions/additional heat treatment and hardness/conductivity of rapidly solidified Al-Fe alloy materials to clarify their mechanisms.

## 2. Materials and Methods

**Figure 2:**
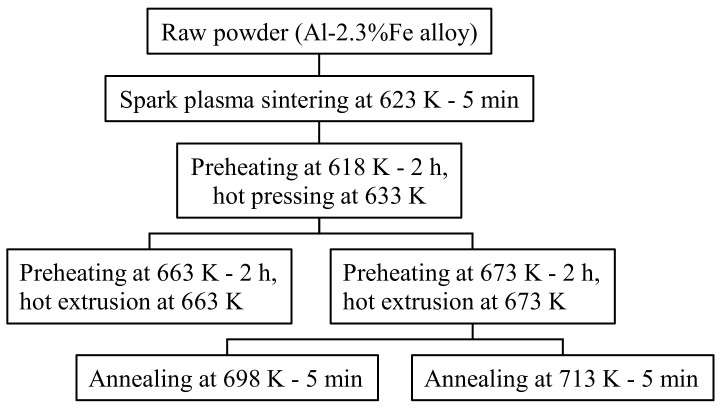
Experimental procedure for sample preparation.

**Figure 3:**
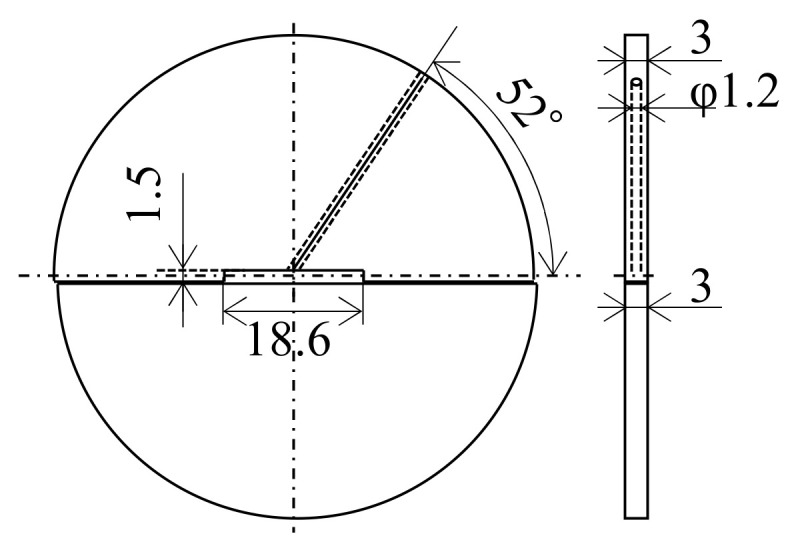
Drawing of the extrusion dies (unit: mm).

The sample preparation method is shown in Figure 2. An air-jet atomized Al-2.3%Fe alloy powder manufactured by Toyo Aluminium K.K. was used as the raw material. The impurities in the alloy are 0.1% or below. The average particle size of the powder is approximately 30 μm. This Al-2.3%Fe alloy powder was spark plasma sintered into a cylindrical body 42 mm in diameter and 23 mm in height under the conditions of a temperature of 623 K and the pressurization of 20 MPa. Four pieces of this sintered body were hot compressed axially in a die with a 42 mm internal diameter at a temperature of 633 K and a load of 1400 kN. This compressed billet was preheated again and then hot extruded into a plate material using a 200-ton horizontal hydraulic press with a container with a 42 mm internal diameter (YKK Corporation). Assuming that the properties of the extrudate would be affected by the phase change from an Al_6_Fe phase to an Al_13_Fe_4_ phase at 623–673 K or above [7], the billet preheating temperature before extrusion was set up in two ways, 663 K and 673 K. The die preheating temperature was set to 773 K, which is higher than the billet preheating temperature because the extruder does not have a function to heat dies. The extrusion die was designed as in Figure 3. We made a hole just above the 18.6 mm-wide bearing surface and inserted a thermocouple into the hole to measure the temperature changes in the die during extrusion (hereinafter referred to as the “in situ die temperature”). The extrusion loads were converted from the hydraulic drive force of the ram. To prevent the material from clogging the extruder, the first 500 mm of the material was extruded at a slow ram speed of 0.5 mm/s, and then, a total approximate length of 2500 mm of the material was extruded at a fast ram speed of 6 mm/s to simulate a steady extrusion. The time taken for the latter steadily extruded portion was about 7 s. Test samples were taken from the 500 mm, 1500 mm, and 2500 mm positions from the front end of the extrudates. In the present paper, these positions are called the “front end,” “center,” and “rear end,” respectively. The relative density of the extrudates (relative to the density of casts) measured by Archimedes’ method was 0.99 or more in all extrusion conditions, which confirmed that the extrudates were completely densified. To understand the effect of the temperature of additional heat treatment on the extrudates, the extrusion samples were annealed in an atmospheric furnace set at 698 K or 713 K for 15 min (soaking for 5 min) and then quenched in water.

The samples were evaluated for hardness by a Vickers hardness test (50 gf in the test load) and for conductivity by a vortex conductometer Sigmatest 2.069 (Foerster Japan Limited, Shinagawa-ku, Japan). The microstructures were observed by FE-SEM (JEOL JSM-7800F) at an accelerating voltage of 5 kV and WD = 10 mm on the raw material powder and the cross-sections parallel to the extrusion direction of the extrudate. The images were binarized and analyzed by ImageJ 1.53 k, which is an open-source image-processing software, to find the area fraction *A*_f_ (equivalent to volume fraction *V*_f_), the average radius of the particles *r*_p_, and the aspect ratio *r*_a_. The radius of the particles was obtained as half the equivalent circle diameter of them. In this analysis, particles below an equivalent circle diameter of 0.05 μm (equivalent to 6 pixels or below) were excluded as noise. To understand the grain structure, the cross-sections parallel to the extrusion direction of the extrudate were observed and analyzed by FE-SEM (ZEISS Gemini 450) and EBSD (Oxford Symmetry). In the analysis, the interfaces with an inclination of 5° or more were considered grain boundaries. To identify the types of the second-phase particles, we conducted an analysis through X-ray diffraction (XRD). For this analysis, synchrotron light (SAGA-LS BL16, 0.0918 nm in wavelength) was used, and the measurement range of 2θ was 5 to 40 degrees.

## 3. Results

**Figure 4:**
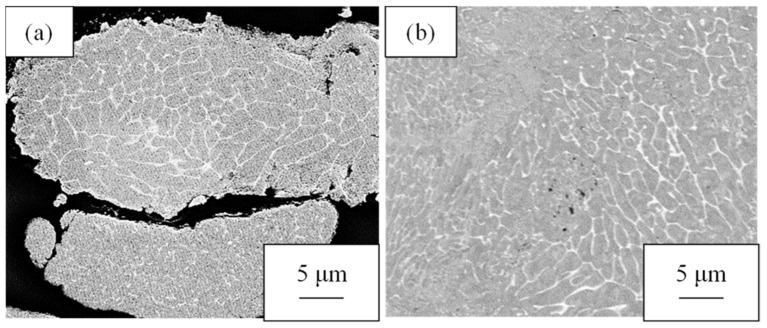
Cross-sectional SEM-COMPO images of (**a**) the Al-2.3%Fe alloy powder and (**b**) a hot-pressed billet made of the powder.

The photos of the cross-sectional structures of the raw material powder and the hot-pressed billet are shown in Figure 4. The cross-sectional structure of the Al-2.3%Fe powder exhibited a mixed structure in cellular and particle dispersion forms consisting of an aluminum matrix with dark contrast and Al-Fe intermetallic compounds with bright contrast. The hot-pressed billet exhibited a mixed structure consisting of cellular and particle dispersion structures, which are the same sizes as those of the raw material powder. This result confirmed that the structural change caused by hot pressing is small.

**Figure 5:**
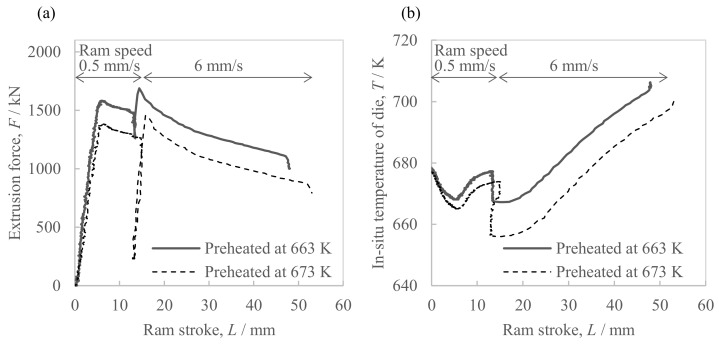
Parameters of the hot extrusion of Al-2.3%Fe alloy billets preheated at 663 K and 673 K\; (**a**) extrusion force and (**b**) in situ temperature of the extrusion die.

Figure 5 shows the changes in extrusion load and the in situ die temperature of the hot extrusion of the Al-2.3%Fe alloy powder billet. At the steady portion at a ram speed of 6 mm/s, the extrusion load in the case of a billet preheating temperature of 663 K was 14% higher than with 673 K. Similarly, the in situ die temperature in the case of a billet preheating temperature of 663 K was approximately 20 K higher than with a preheating temperature of 673 K. With both billet preheating temperatures, the in situ die temperature at the steady portion at a ram speed of 6 mm/s increased by 40–50 K or so when the ram strokes were increased.

**Figure 6:**
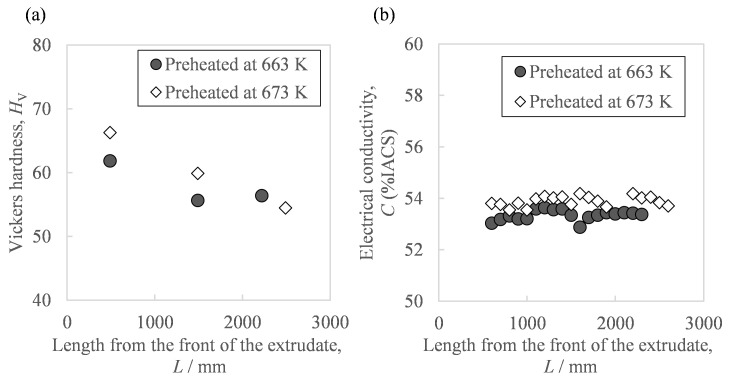
Relationships between the properties of the Al-2.3%Fe alloy extrudate and the distance from the front of the extrudate: (**a**) Vickers hardness and (**b**) electrical conductivity.

**Figure 7:**
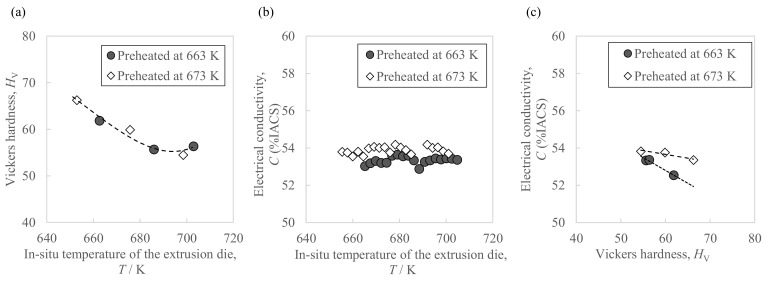
Relationships between the properties of the Al-2.3%Fe alloy extrudate and the in situ temperature of the extrusion die: (**a**) Vickers hardness, (**b**) electrical conductivity, and (**c**) their correlation.

Figure 6 shows the hardness and conductivity of the extrudate. As in Figure 6a, the front end showed a higher Vickers hardness than the extrudate’s center and rear end. Moreover, as shown in Figure 6b, there were a few differences in conductivity due to the sampling position except for small fluctuations from measurement errors. The average conductivity in the case of the higher billet preheating temperature of 673 K was 0.6 points higher than with 663 K. In addition, from the relationship between the extrusion progress and the in situ die temperature (Figure 5), we also found the relationship between the sampling position and in situ die temperature. Figure 7 shows the relationship between the extrudate properties and the in situ die temperature. As in Figure 7a, the relationship between the extrudate hardness and the in situ die temperature at billet preheating temperatures of 663 K and 673 K was found on one curve, where the hardness tended to decrease as the in situ die temperature increased. Moreover, after the in situ die temperature exceeded 690 K, the hardness seemed to increase slightly. As in Figure 7b, the in situ die temperature had little effect on the conductivity. As in Figure 7c, compared to the same hardness value of about 60 HV, the electrical conductivity of the sample preheated at 673 K was about 1 point higher than that at 663 K. The preheating temperature was confirmed to be effective in changing the electrical conductivity.

**Figure 8:**
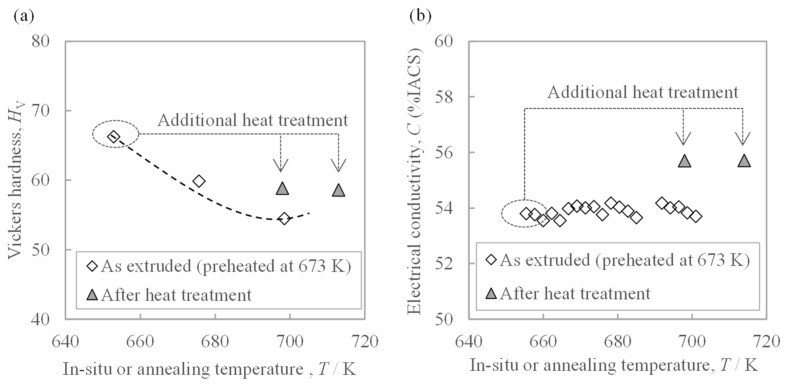
Relationship between the in situ temperature of the extrusion die or additional heat treatment temperature and (**a**) hardness and (**b**) electrical conductivity of the extrudate, which was preheated at 673 K before extrusion. The dotted line in (**a**) is the trend line found in Figure 7a.

The relationships between the temperatures of additional annealing on the extrudate, the in situ die temperature, and properties are jointly shown in Figure 8. Additional annealing decreased the hardness and increased the conductivity. Comparing the materials exposed to the same temperature of 700 K, the hardness of the as-extruded material was lower than that of the heat treated material, and the electrical conductivity was also lower. Thus, the effect of increasing the in situ die temperature during extrusion on the hardness was revealed to be different from that of the subsequent heat treatment.

**Figure 9:**
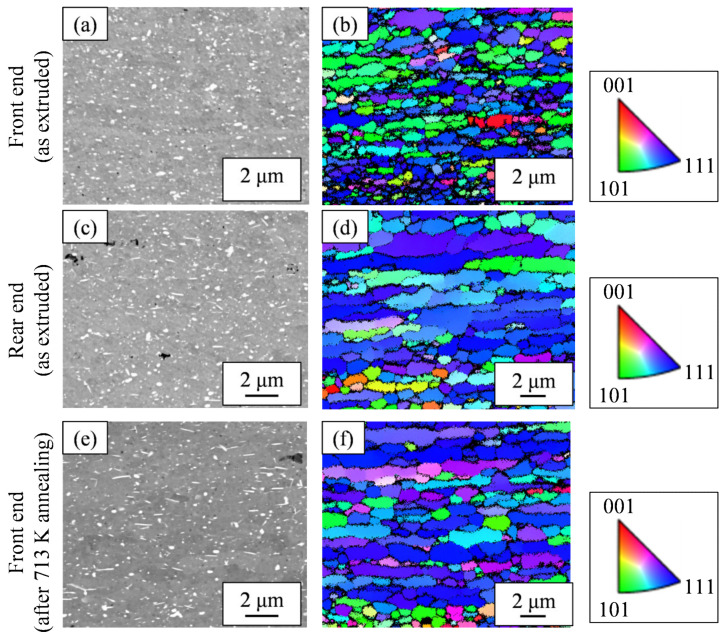
Microstructures of the Al-2.3%Fe alloy extrudates which were preheated at 673 K before extrusion: (**a**,**c**,**e**) SEM-compo images and (**b**,**d**,**f**) EBSD-IPF images. Note that the observed areas of the SEM-compo images and EBSD images are different.

**Figure 10:**
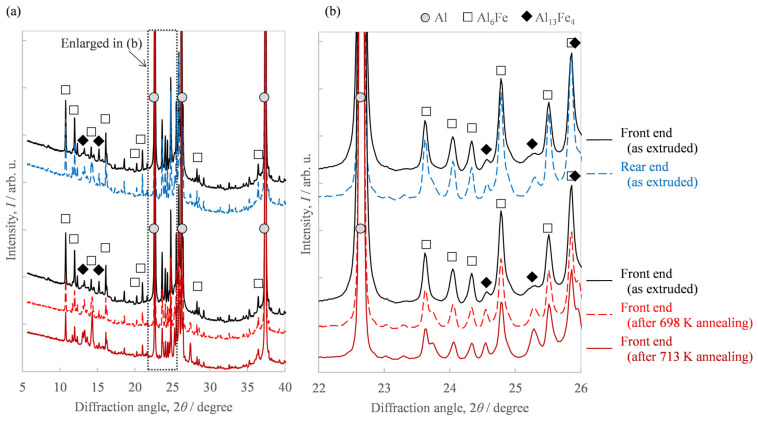
XRD profile of the Al-2.3%Fe alloy extrudate, which was preheated at 673 K before extrusion: (**a**) full 2*θ* range of the measurement and (**b**) enlarged 2*θ* range.

Of the extrudates with billet preheating temperatures of 663 K and 673 K, Figure 9 shows the microstructures of both the front end and rear end in the case of a preheating temperature of 673 K, which showed a greater fluctuation in hardness in Figure 6a. While the second-phase particles at the front end in Figure 9a were spherical, the rear end in Figure 9c exhibited somewhat coarse needle-like particles in addition to spherical ones. From the EBSD-IPF images in Figure 9b,d, we found the area weighted average of the grain size. The grain size of the front end was 1.2 μm and that of the rear end was 3.8 μm, indicating that the grain size of the rear end was coarser than that of the front end. Figure 9e shows the microstructural photo of the front end after annealing at 713 K, which indicates the amount of needle-like particles increased compared to Figure 9a. The grain size of the front end in Figure 9f was 2.2 μm. Figure 10 shows the results of the synchrotron light XRD analysis. As another second phase of the aluminum matrix, a peak corresponding to the metastable Al_6_Fe phase was observed in addition to the stable Al_13_Fe_4_ phase. The peak intensity of the Al_13_Fe_4_ phase at the rear end was higher than that at the front end. The additional heat treatment somewhat weakened the diffraction peak intensity corresponding to the Al_6_Fe phase and, at the same time, increased the peak intensity of the Al_13_Fe_4_ phase.

## 4. Discussion

### 4.1. The Effect of Temperature Change during Extrusion on the Microstructure, Hardness, and Conductivity

**Figure 11:**
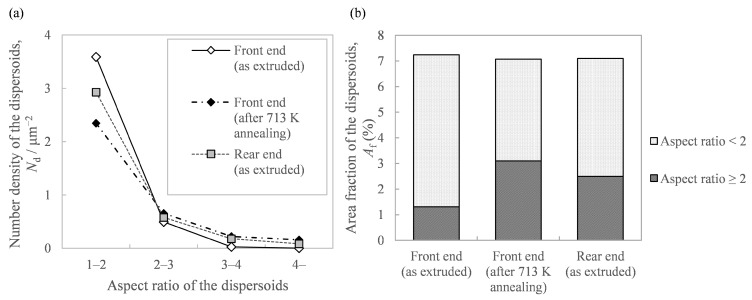
Analysis of the Al-Fe dispersoids’ morphology in Figure 9: (**a**) histogram of the aspect ratio of the Al-Fe dispersoids and (**b**) area fraction of the Al-Fe dispersoids classified with the aspect ratio.

Regarding the effect of the in situ die temperature and additional annealing temperature on the microstructure, we identified that the increase in these temperatures resulted in coarser needle-like second-phase particles (Figure 9). Figure 11a shows the histograms of the aspect ratios of the Al-Fe dispersoids in Figure 9. Compared to the as-extruded front end, the other samples tended to have more particles with a large aspect ratio. Figure 11b shows the area fraction *A*_f_ of the dispersoids classified with an aspect ratio of 2 as the threshold. While the total area fraction of the Al-Fe dispersoids did not change, the fraction of particles with a large aspect ratio was smaller in the front end than in the other samples. These results suggest that when Al-Fe extrudates are exposed to high temperatures by additional heat treatment or by increasing the in situ temperature, the low aspect ratio dispersoids transformed into large ones. Additionally, we identified that the increase in these temperatures raised the peak intensity of the Al_13_Fe_4_ phase in the XRD profile, as in Figure 10. These structural changes led us to suppose that the phase change occurred from the spherical Al_6_Fe phase to the needle-like Al_13_Fe_4_ phase as a result of the increase in temperature. Wu [7] reported that spherical fine Al_6_Fe transforms into coarse rod-shaped Al_13_Fe_4_ in a rapidly solidified Al-2%Fe alloy through heat treatment above 623 K, which is consistent with that supposed in the present study. According to the report [7], the Al_6_Fe phase changes directly to the Al_13_Fe_4_ phase without undergoing any intermediate phase, and therefore in our experiment, it is considered that the phase change proceeded within a short extrusion time of approximately 7 s.

**Figure 12:**
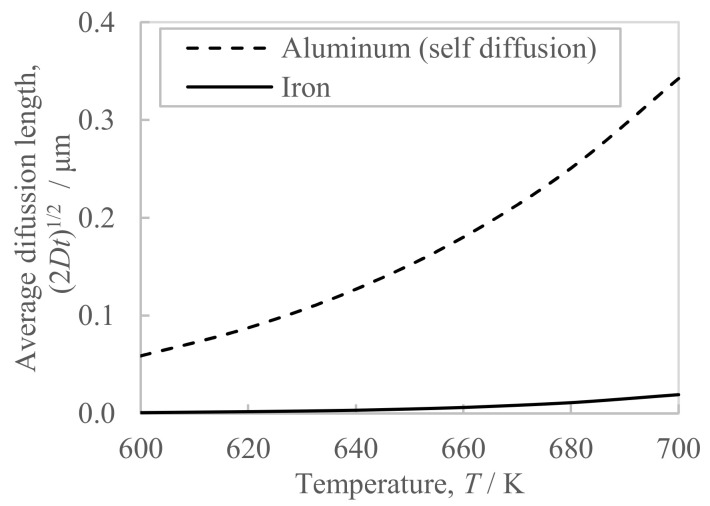
Average diffusion length in the aluminum matrix of the aluminum and iron at 600–700 K for 7 s, where *D* is the diffusion coefficient, and *t* is the diffusion time. The parameters for the calculation of the diffusion coefficient *D* were cited from the reference [12].

The conductivity of the extrudate with the higher billet preheating temperature of 673 K turned out to be 0.5 point higher than in the case of the lower preheating temperature of 663 K (Figure 7b). This conductivity difference suggests that the billet preheating temperature affected the amount of iron solute because the lower the amount of iron solute, the higher the conductivity of the Al-Fe alloy becomes [11]. Generally, the amount of iron solute contained in rapidly solidified Al-Fe alloy powder exceeds the equilibrium state, which is approximately 0.05% [7]. When this powder is heated to 523 K or above, the Al-Fe intermetallic compounds precipitate [7] and the amount of iron solute in the matrix decreases. Thus, the higher the billet preheating temperature, the more Al-Fe compounds precipitate from the iron solute supersaturated in the raw material powder, and therefore, it is thought that the amount of iron solute decreased and accordingly the conductivity improved. On the other hand, the increase in the in situ die temperature did not affect the conductivity of the extrudate (Figure 7b). One of the factors is considered to be the short period of exposure of approximately 7 s to the increase in the in situ temperature during extrusion, which is shorter than the billet preheating time of 2 h. It is known that the diffusion length of iron in aluminum is significantly slower than that of other elements except for transition metals [12]. Figure 12 shows the calculated diffusion length in the aluminum matrix based on the parameters in the reference [12]. The diffusion length of iron during the 7 s required for hot extrusion is significantly smaller than the self-diffusion of aluminum. Therefore, we believe that the nucleation and growth of new Al-Fe compounds did not progress in the short time of 7 s, making it difficult for the amount of iron solute to decrease. Moreover, the additional heat treatment at 698 K and 713 K increased the conductivity of the extrudate by approximately two points (Figure 8b). It is considered that the precipitation of Al-Fe compounds proceeded during the soaking time of this additional heat treatment, which takes approximately 5 min and is longer than the extrusion time of approximately 7 s. Therefore, it has been made clear that the conductivity of the extrudate is changed by high-temperature heating for several minutes or more, for example, by preheating the billet before extrusion or heating after extrusion, and the higher the temperature, the higher the conductivity becomes.

### 4.2. Mechanism of Hardness Change with In Situ Die Temperature Increase and Additional Heat Treatment

We compared the contributions of the strengthening factors for rapidly solidified Al-Fe alloys (work hardening, solid solution strengthening, particle dispersion strengthening, and grain refinement strengthening) in order to clarify the mechanism by which the structural change due to the increase in the in situ die temperature and additional heat treatment causes the hardness to decrease.

Since the extrudate and the additionally heat-treated extrudate were fully recrystallized in the structure (Figure 9), the contribution of work hardening can be ignored.

Regarding the solid solution strengthening by iron, because there is little difference in conductivity in the longitudinal direction of the extrudate as an index of the amount of solute (Figure 6), the effect of solid solution strengthening can be ignored as a factor of the hardness decrease caused by the increase in the in situ die temperature. As for the conductivity increase of approximately two points due to additional heat treatment (Figure 8b), the corresponding difference in the amount of iron solute (%) is only 0.05 points [11,13]. With regard to this, Nayak [14] reported that the hardness increased from 0.89 GPa to 1.43 GPa (equivalent to 91 HV to 146 HV, respectively) as the amount of added iron in the rapidly solidified Al-Fe alloy increased from 2.5% to 5% atomically (equivalent to 5% to 9.8% by mass, respectively). If it is hypothesized that all the added iron contributed to the solid solution strengthening in the report [14], the increase in hardness with a one-point increase in the amount of iron solute will be around 12 HV. Hence, in a case where the amount of solute (%) is reduced by 0.05 points, the change in hardness is considered to be around 0.6 HV, which is small compared to the hardness reduction of approximately 7 HV caused by additional heat treatment; therefore the effect of solid solution strengthening as a factor can be ignored.

Next, the contribution of dispersion strengthening was discussed by using the microstructural features found in Figure 9. The parameters for the analysis are summarized in Table 1, where *r*_p_ is half the value of the average equivalent circle diameter *d*_p_ of dispersoids (*r*_p_ = *d*_p_/2). Assuming the Orowan strengthening mechanism by fine dispersoids, the strengthening contribution by fine Al-Fe dispersoids $\Delta \sigma_{0.2}$ would be roughly evaluated by [15]:

(1) $$\Delta \sigma_{0.2}\propto V_{f}^{1/2}\cdot r_{p}^{-1}$$

**Figure 13:**
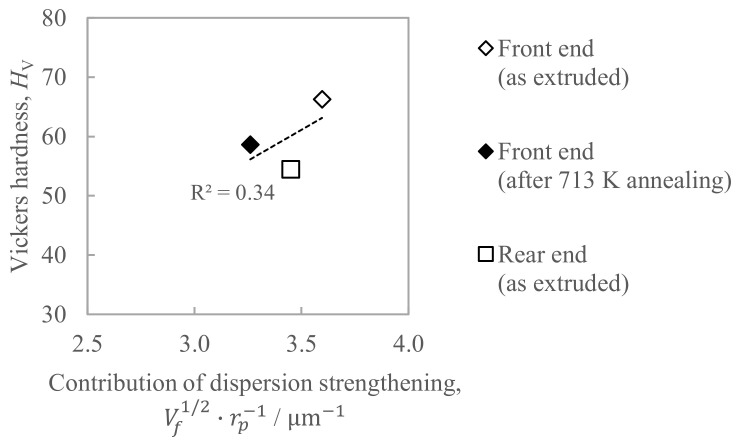
Relationship between the hardness and the contribution of the dispersion strengthening estimated by Equation (1).

Figure 13 shows the relationship between the hardness and the contribution of dispersion strengthening, where the right sides of Equation (1) was employed as an index. The coefficient of determination *R*^2^ in Figure 13 was as small as 0.34, which indicates that dispersion strengthening is not considered to be a controlling factor for hardness.

The contribution of grain refinement strengthening $\Delta \sigma_{0.2}$ was estimated from the Hall–Petch law of the following equation:

(2) $$\Delta \sigma_{0.2}\propto d_{G}^{-1/2}$$

**Figure 14:**
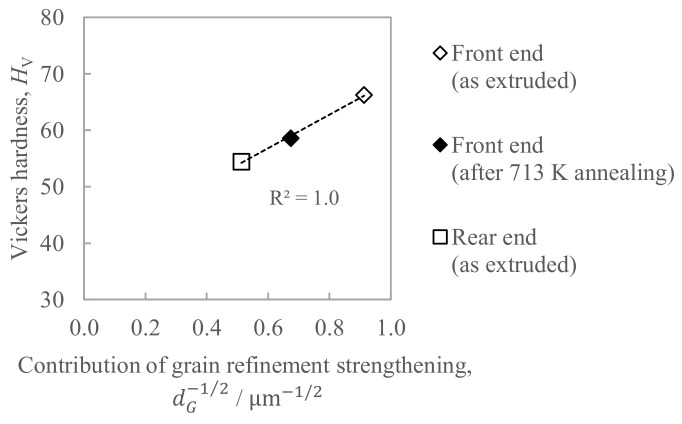
Relationship between the hardness and the contribution of the grain refinement strengthening estimated by Equation (2).

Figure 14 shows the relationship between the hardness and the contribution of grain refinement strengthening, where the right sides of Equation (2) are employed as an index. Since the coefficient of determination *R*^2^ in Figure 14 was sufficiently large, with it being as much as 1.0, the determinant of hardness is considered to be grain refinement strengthening. Hence, it is considered that the hardness decrease with the increase in the in situ die temperature is caused by the coarsening of the grain size.

### 4.3. Causes of Grain Coarsening

**Figure 15:**
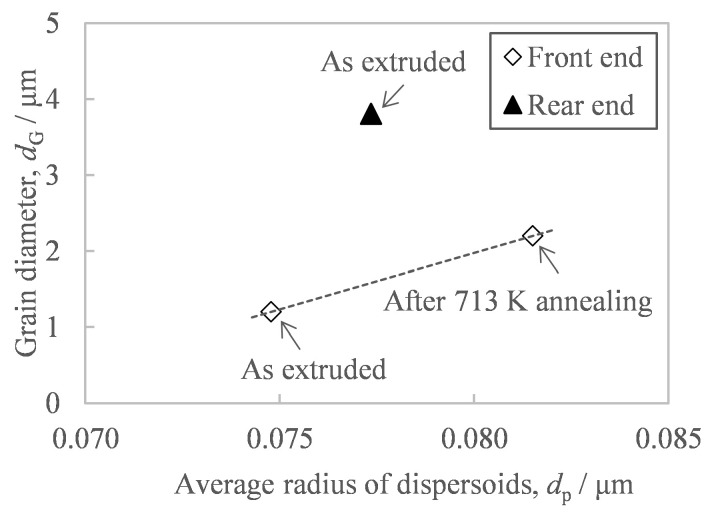
Relationship between the average diameter of second-phase particles and the grain diameter of the aluminum matrix of the extrudate preheated at 673 K before extrusion.

In order to suppress the hardness fluctuation of extrudates, our study has revealed that it is necessary to suppress the coarsening of the grain size due to the temperature increase during extrusion. It is well known that when fine Al-Fe compounds are dispersed, the grain boundaries of the aluminum matrix are pinned by the dispersoids and are therefore hardly move even when exposed to high temperatures of 523–573 K [15,16]. Since this pinning force depends on the size of the Al-Fe dispersoids, the crystal grain size of the aluminum matrix tends to be proportional to the size of the dispersoids [16]. However, there are no reports so far on the relationship between grain size and in situ temperature in hot working. Figure 15 shows the relationship between the average radius of Al-Fe dispersoids and the grain size of the aluminum matrix. Comparing the samples at the front end, there is a positive correlation between the grain diameter and the average radius of dispersoids, which is consistent with previous reporting [16]. However, the grain size at the rear end, where the in situ temperature of hot extrusion increased, was found to be much larger than the trend line for samples at the front end. These results show that an increase in the in situ temperature during hot working made the grain size larger than an increase in the temperature during heat treatment. This difference may be due to the difference in the magnitude of accumulated strain energy, which is the driving force of grain boundary migration. This is because the in situ strain energy accumulated during hot extrusion is greater than the strain energy remaining after extrusion.

**Figure 16:**
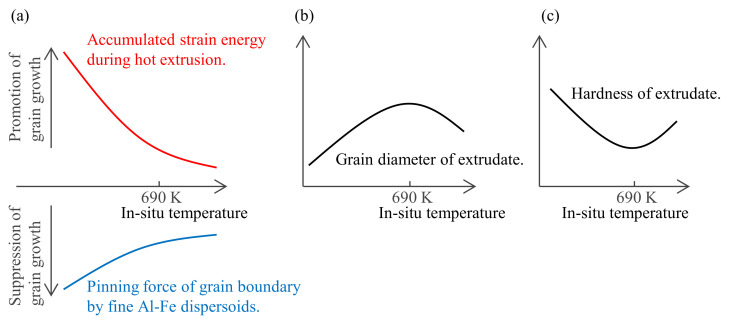
Schematic illustration of the mechanism by which the in situ extrusion temperature affects hardness: (**a**) the balance between the driving force of grain boundary migration and the pinning force, (**b**) resultant grain diameter, and (**c**) the hardness of the extrudates.

The relationship between hardness and the in situ temperature of the extrusion die followed a curve with a minimum around 690 K (Figure 7a). The assumed material factor is the balance change between the driving force of grain boundary migration and the pinning force. Generally, the higher the hot working temperature of aluminum is above 623 K, the more likely that dynamic recovery occurs and the strain accumulated in the matrix decreases [17]. On the other hand, it has been confirmed that the higher the in situ extrusion temperature is above 650 K, the more the second-phase particles coarsen and the pinning force of the grain boundary migration decreases (Figure 9). As illustrated in Figure 16, these effects of suppressing grain growth (due to a decrease in accumulated strain energy) and promoting grain growth (due to a decrease in pinning force) might be balanced around 690 K. As a possible experimental method to test this hypothesis, the accumulated strain can be changed while keeping the in situ temperature of extrusion constant and suppressing the size change of the second-phase particles, since not only temperature but also the strain rate affects the amount of accumulated strain in aluminum [17]. Therefore, in order to clarify the mechanism by which the minimum hardness was around 690 K, a systematic study on the mutual change in extrusion rate and in situ die temperature will be needed in the future.

**Figure 17:**
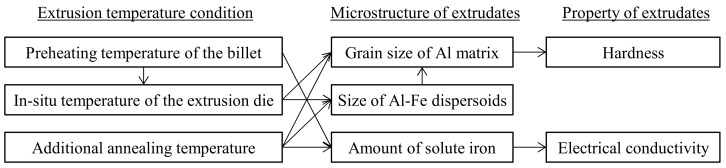
The effect of the extrusion conditions on the material structure and properties.

The relationship between the extrusion temperature conditions, microstructures, and properties of the extrudates, as discussed thus far, is summarized in Figure 17. One of the remarkable results of the present study is the clarification of the effect of the in situ temperature of the extrusion die on the hardness of the extrudates. When the in situ temperature rises, the Al-Fe dispersoids and grain of the aluminum matrix become coarse, resulting in a decrease in hardness. As for the electrical conductivity, it was clarified that if the heat treatment time is several minutes or longer, such as with preheating or additional annealing, the amount of solute iron decreases, resulting in the electrical conductivity increasing. These findings are important for the practical application of a rapidly solidified Al-Fe alloy produced by hot extrusion.

## 5. Conclusions

We studied the relationship between the extrusion temperature conditions/additional heat treatment and hardness/conductivity of a rapidly solidified Al-Fe alloy and obtained the following findings.

1. The hardness of an extrudate is governed by the in situ temperature during extrusion; the hardness decreases the higher the in situ temperature is above 650 K and is at the minimum at around 690 K. Moreover, the hardness of the extrudate decreases with additional heat treatment after extrusion. The decrease in the hardness is caused by the coarsening of the grain size of the aluminum matrix, which is caused by the coarsening of Al_6_Fe phase particles and the generation of coarse Al_13_Fe_4_ phase particles and the resultant decrease in the pinning force of the aluminum matrix grain boundary.
2. The higher the billet preheating temperature before extrusion or the temperature of additional heat treatment after extrusion is, the more the conductivity of the extrudate increases. This increase in the conductivity that is believed to be achieved by the decrease in the amount of iron dissolved in the aluminum matrix requires a few minutes or more because the diffusion and the precipitation of iron in aluminum is slow.

## Figures and Tables

**Table 1:** Parameters for analysis of the contribution of strengthening mechanisms. The microstructural features were analyzed from the SEM-compo and SEM-EBSD images in Figure 9.

Sample	Vickers Hardness *H*_V_	Al-Fe Dispersoids		Aluminum Matrix
		Volume Fraction *V*_f_	Average Radius *r*_p_	Grain Diameter *d*_G_
Front end (as extruded)	66	7.2%	0.075 μm	1.2 μm
Front end (after 713 K annealing)	59	7.1%	0.082 μm	2.2 μm
Rear end (as extruded)	54	7.1%	0.077 μm	3.8 μm

## Data Availability

Not applicable.
